# A Longitudinal Study of BCG Vaccination in Early Childhood: The Development of Innate and Adaptive Immune Responses

**DOI:** 10.1371/journal.pone.0014066

**Published:** 2010-11-19

**Authors:** Yenny Djuardi, Erliyani Sartono, Heri Wibowo, Taniawati Supali, Maria Yazdanbakhsh

**Affiliations:** 1 Department of Parasitology, Faculty of Medicine, University of Indonesia, Jakarta, Indonesia; 2 Department of Parasitology, Leiden University Medical Center, Leiden, The Netherlands; Institut Pasteur, France

## Abstract

BCG vaccine drives a strong T helper 1 cellular immunity which is essential for the protection against mycobacteria, however recent studies suggest that BCG vaccination can have non-specific beneficial effects unrelated to tuberculosis. In the present cohort study the development of cytokine profiles following BCG vaccination was investigated. Immune responses to PPD were assessed before vaccination and at ages of 5 months, 1 year, and 2 years, followed by BCG scar measurement at 4 years of age. BCG was shown to induce both Th1 and Th2 type responses against PPD at about 5 months of age after vaccination, and while Th1 response was sustained, Th2 responses declined over time. However, BCG scar size was strongly correlated with Th2 responses to PPD at 5 months of age. Importantly, we observed no clear effects of BCG vaccination on innate immune responses in terms of early IL-10 or TNF-α production whereas some alterations in general adaptive immune responses to PHA were observed.

## Introduction

Most immunological studies on the efficacy of Bacille Calmette-Guérin (BCG) vaccination focus on the production of IFN-γ as the main feature of Th1 response, which leads to the activation of important cells such as macrophages to contain mycobacteria. Besides the partial protection against TB through IFN-γ production [1], BCG may decrease the mortality and morbidity in childhood and adulthood by its non-specific effects on the immune system [2], [3]. The presence or absence of BCG scar has been used as one of the indicators for successful vaccination [4], while not necessarily correlated with protection against tuberculosis [5], studies in Guinea Bissau have shown better survival and less respiratory infections in children with BCG scars [6], [7]. These observations on the impact of BCG has been proposed to be caused by the enhancement of the maturation of the innate and adaptive immune responses [8]. However, very few studies have examined whether BCG vaccination in childhood alters not only responses to mycobacterial antigens, but also to mitogens or to stimuli of the innate immune system. Moreover, environmental factors can affect neonatal immune responses, influencing both specific and non-specific immune reactivities. Factors such as living on traditional farms or parasitic infections are known to affect the immune system in early life with possible consequences for disease outcome later in life [9], [10] or for responses to vaccination at infancy [11]. Indeed, it is known that chronic helminth infection can modulate immune responses of the host to produce more Th2 and regulatory cytokines against helminth and bystander antigens. In developing countries, infants can be exposed to helminth antigens from early life, even in utero, which may affect the child's subsequent immune responses to Th-1 producing vaccines such as BCG [11], [12]. There are so far very few studies that have examined the development and progression of cellular immune responses following BCG vaccination of neonates over time and the effect that environmental factors may have on this process.

In the current longitudinal study following BCG vaccination of neonates in Indonesia, we have examined the production of Th1 (IFN-γ) and Th2 (IL-5, IL-13) cytokines in day 6 supernatants of whole blood in response to PPD and PHA to determine specific and non-specific adaptive immune responses. To assess the effect of BCG vaccination on the development of innate immune responses, IL-10 and TNF-α were measured in one day culture supernatants of whole blood stimulated with LPS and PPD. We also analyzed the relationship between cytokine responses to mycobacterial antigens and scar formation at an age when the scar formation has stabilized. In order to assess how external factors might influence responses to BCG vaccination, we studied the effect of maternal parasitic infection status on the profile of cytokine production over time.

## Methods

### Ethics Statement

This study was conducted according to the principles expressed in the Declaration of Helsinki. The study was approved by Ethics Committee of Faculty of Medicine, University of Indonesia. All mothers provided written informed consent for the collection of samples from their children and for subsequent analysis.

### 2.1. Blood collection, BCG vaccination and measurement of BCG scar

This study was performed as part of a birth cohort to examine the development of immune responses of children living in areas endemic for helminth infection. Maternal parasitological data such as filarial or intestinal parasite infection was obtained during the recruitment of pregnant women as described before [13]. Filarial antigenemia for *Wuchereria bancrofti* was determined by immunochromatographic test (ICT) as described by the manufacturer (Binax, Scarborough, ME, USA). The presence of intestinal helminth eggs and protozoa cysts was determined from direct stool examination by microscopy, using lugol staining.

Children were recruited from two adjacent villages in a peri-urban area, after their mothers gave informed consents for participation in this study. Blood was withdrawn by venipuncturist and was collected in a heparinized tube at several time points: T0: before BCG vaccination, T5: 5 months of age, T12: 1 year of age, T24: 2 years of age. In Indonesia, BCG vaccination is the first vaccine to be given according to the national vaccination program, followed by three Hepatitis B vaccination, three diphtheria-pertusis-tetanus (DPT) that are given together with three oral polio vaccination, and finally measles vaccination before the child reaches 1 year of age. BCG vaccination program requires every infant to be vaccinated soon after birth by health staff from local primary health care center (PHC). BCG vaccine used here contains attenuated live *Mycobacterium bovis* strain Paris No. 1173-P2 (Biofarma, Bandung, Indonesia), and 0.05 ml is given by intradermal injection of the arm.

After BCG vaccination, the resulting BCG scars were measured at 4 years of age by the same person from the research team, and the mean diameter of scar size was calculated from diameters perpendicular to each other. The reason for measuring BCG scar at 4 years of age is because the scar formation is expected to have stabilized. A BCG scar was considered negative if the mean diameter was less than 2 mm.

### 2.2. Whole blood culture

Heparinized venous blood was processed for culture within 6 hours after venipuncture. As previously described [14], the whole blood was diluted 10 times in RPMI 1640 medium (Invitrogen, Breda, The Netherlands) and was stimulated with tuberculin purified protein derivative of *Mycobacterium tuberculosis* (PPD) batch RT 50 (10 µg/ml; Statens Serum Institut, Denmark, Copenhagen), phytohaemagglutinin (PHA; 2 µg/ml; Wellcome Diagnostics, Dartford, UK), lipopolysaccharide (LPS; 100 ng/ml; Sigma-Aldrich chemie, Zwijndrecht, The Netherlands) or medium only as a negative control. One hundred microlitres of stimuli were added to each well containing 100 µl diluted blood in a round-bottomed 96 well plates (Nunc, Roskilde, Denmark). LPS stimulation was performed in 101 from 147 samples (69%). The plates were incubated in the presence of 5% CO2, at 37°C. Supernatants were collected on day 1 for interleukin (IL)-10 and TNF-α measurement to assess innate immune responses to LPS and PPD, and on day 6 for IL-5, IL-13, IFN-γ measurement to discern antigen specific (PPD) and non-specific polyclonal (PHA) adaptive immune responses. All supernatants were kept frozen in −20°C until measurement. The cytokine measurements were performed by multiplex bead-based assay as described below.

### 2.3. Cytokine measurement in supernatants

IL-10, TNF-α, IL-13 and IFN-γ levels were measured in supernatants by in-house multiplex bead based assay using Luminex IS 100 (Luminexcorp, Austin, TX, USA) while IL-5 level was measured by ELISA as described elsewhere [13]. The detection limits for IL-10, TNF-α, IL-13, IFN-γ and IL-5 were 6.5 pg/ml, 1.7 pg/ml, 12.5 pg/ml, 3.6 pg/ml, 2 pg/ml, respectively. All samples with values below detection limit were given half of these threshold values.

### 2.4. Statistical analyses

Cytokine levels were analyzed in two ways, either using raw cytokine levels when comparing cytokines between two groups of children or using net cytokine levels (substracted from the negative control) to obtain percentage of responders/non-responders (above or below zero for substracted values). Mann-Whitney non- parametric test was used to compare cytokine levels which were not normally distributed. Since the sample size in each time point was not equal, Mann-Whitney test was used to compare cytokine responses between two time points of measurement. The correlations between cytokine responses to PPD and PHA and between BCG scar size and cytokine responses were analyzed using Spearman's rank correlation. In order to adjust cytokine levels for other variables (gender, birth weight, birth season, age receiving BCG, place of residence, maternal intestinal helminth or protozoan infection), multiple linear regression was performed with diameter of BCG scar as the outcome. Age receiving BCG, diameter of BCG scars and cytokine levels were previously log-transformed to get a normal distribution. The statistical analyses were performed in SPSS version 16. A two-tailed *p* value was considered significant if less than 0.05.

## Results

### 3.1. Study subjects

A total of 147 children were recruited into the study and blood samples were obtained before BCG vaccination was given (T0). From these children, 120 children could be followed up at T5, 105 children at T12 and 98 children at T24. Apart from 66 children in whom blood samples were available at all time points, there were 81 children in whom blood samples were not available at one or two follow up time points due to the refusal of parents at the particular time point to allow blood sampling of their child (*n* = 14), child discomfort (crying) (*n* = 2), moving out of the study area (*n* = 5), death of the child (*n* = 1), not able to find the child and family (*n* = 37), and lack of sufficient blood for whole blood culture (*n* = 22). There were 6 infants who had received BCG with unclear dates but before any other vaccination; therefore they were included in the analysis. From 141 children with known date of BCG vaccination, the average age at which BCG vaccination was given was at 5 weeks (IQR = 2.0–8.5). Of these, 4 infants (3%) received BCG at the age of less than 1 week, 63 infants (44%) at the age between 1 week and 4 weeks, 39 infants (28%) at the age between 4 weeks and 8 weeks, and 35 infants at the age more than 8 weeks (25%). The mean interval time between BCG vaccination and blood collection at 5 months of age was 20.1 weeks (SD = 5.8 weeks). As shown in Table 1, the child's characteristics were similar between those with complete and incomplete cytokine data. For the comparison of cytokine production before and after BCG vaccination, we used the data from all children (n = 147), while for the relationship between cytokine data and BCG scar we analyzed data from children with complete cytokine results (n = 66).

**Table 1:** Comparison of child's characteristics with BCG scar measurement and cytokine data at all time points (complete data), children with incomplete cytokine data and all children recruited into the study.

Child characteristics	All children (n = 147)	Children with complete cytokine data (n = 66)	Children with incomplete cytokine data (n = 81)
**Place of residence**			
Jati Sampurna	69 (47%)	34 (52%)	35 (43%)
Jati Karya	78 (53%)	32 (48%)	46 (57%)
**Mean birth weight (kg) (SD)**	3.2 (0.5)	3.2 (0.4)	3.2 (0.5)
<3 kg	23/99 (23%)	13/50 (26%)	10/49 (20%)
> = 3 kg	76/99 (77%)	37/50 (74%)	39/49 (80%)
**Birth season**			
dry season	74 (50%)	39 (59%)	35 (43%)
rainy season	73 (50%)	27 (41%)	46 (57%)
**Gender**			
male	70/141 (50%)	36/66 (54%)	34/75 (45%)
female	71/141 (50%)	30/66 (46%)	41/75 (55%)
**Median age at BCG vaccination (week) (IQR)**	5 (2.0–8.5)(n = 141)	4 (1.7–8.2)(n = 66)	5 (2.0–9.0)(n = 75)
**Mean interval BCG vaccination and T5 (week) (SD)**	20.1 (5.8) (n = 119)	20.7 (5.7)(n = 66)	19.4 (5.9) (n = 53)
**Median BCG scar at 48 months (mm) (IQR)**	3.3 (2.0–4.5) (n = 96)	3.3 (1.5–4.1) (n = 58)	3.5 (2.4–4.8) (n = 38)
**Maternal parasitic infections**			
Filaria	35/147 (24%)	15/66 (23%)	20/81 (25%)
Intestinal helminth infection	51/139 (37%)	18/65 (28%)	33/74 (45%)
*Hookworm*	25/139 (18%)	8/65 (12%)	17/74 (23%)
*Ascaris lumbricoides*	21/139 (15%)	9/65 (14%)	12/74 (16%)
*Trichuris trichiura*	11/139 (8%)	4/65 (6%)	7/74 (9%)
Intestinal protozoan infection	39/139 (28%)	13/65 (20%)	26/74 (35%)
*Blastocystis hominis*	31/139 (22%)	10/65 (15%)	21/74 (28%)
*Others* *	15/139 (11%)	3/65 (5%)	12/74 (16%)
Co-infection of intestinal helminth and protozoan	20/139 (14%)	6/65 (9%)	14/74 (19%)

*other intestinal protozoa species: *Entamoeba histolytica/dispar, E. coli, Iodamoeba bütschlii, E. nana, E. hartmani*.

### 3.2. Adaptive immune responses before and after BCG vaccination: Th1 and Th2 cytokine production in response to PPD and PHA

**Figure 1:**
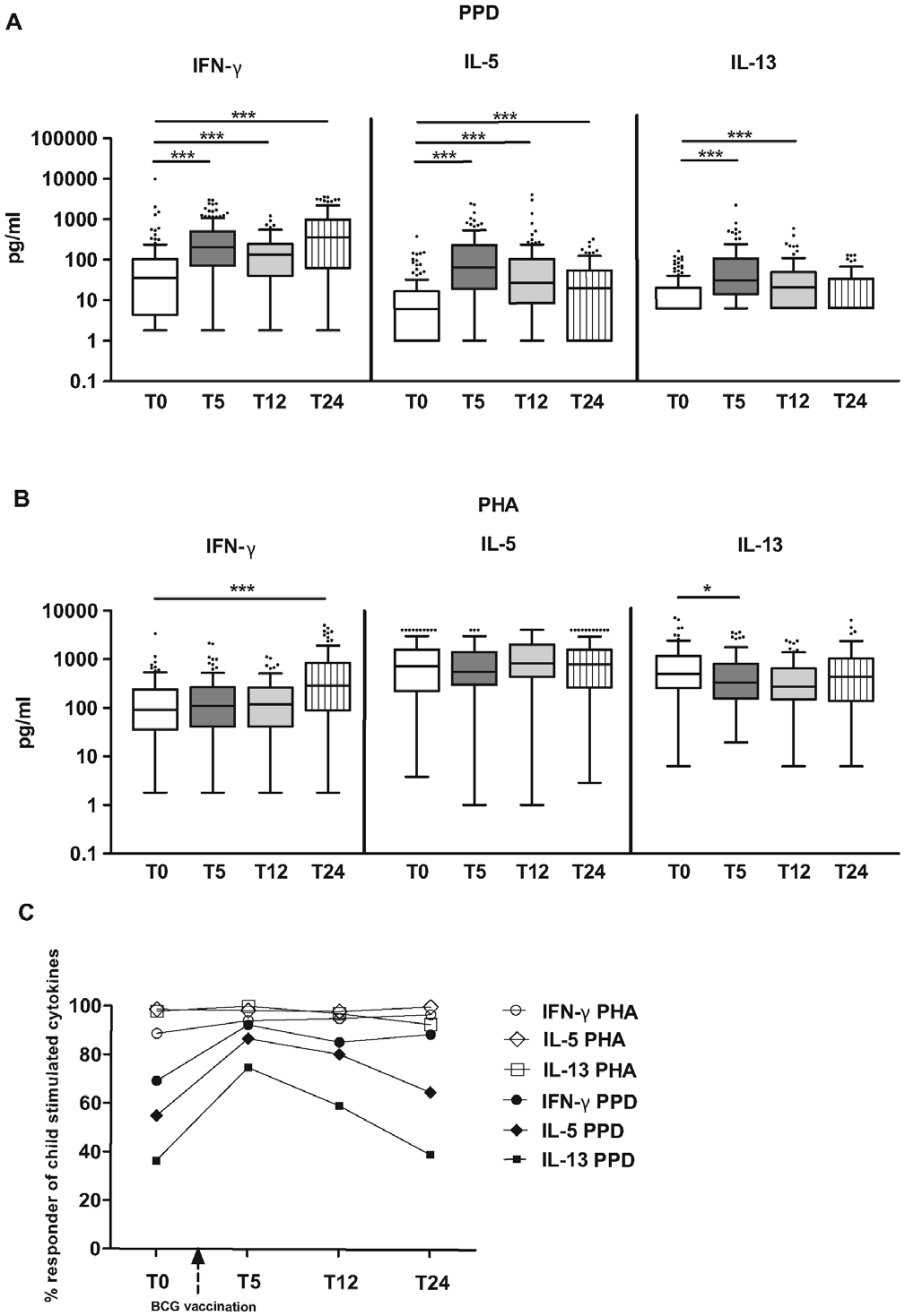
Adaptive cytokine responses to PPD and PHA before and after vaccination. IFN-γ, IL-5, IL-13 responses to PPD (A) and PHA (B). Levels of cytokines at T0 (before vaccination, n = 143); T5 (5 months of age, n = 119), T12 (1 year of age, n = 103) and T24 (2 years of age, n = 97).The horizontal lines in the bars represent the median, with the lower 25% and upper 75% percentiles and extended whiskers the 10% and 90% percentiles. Mann-Whitney test: *0.05>*p*≥0.01; **0.01>*p*≥0.001; ***0.001>*p*. C: Percentage of PPD and PHA responders. T0: before BCG vaccination, T5: at 5 months of age, T12: at 1 year of age, and T24: at 2 years of age. A responder in terms of cytokine production in response to a stimuli is defined as producing cytokine after stimulation above the background production of cytokine when no stimulus is added (level in a stimulated culture above zero after substraction of background cytokine level).

In comparison with pre vaccination time point (T0), IFN-γ, IL-5 and IL-13 responses to PPD increased after BCG vaccination at 5 months of age (p<0.001 for all cytokines). In contrast to IFN-γ which remained high until the children reached 2 years of age, IL-5 and IL-13 responses showed a gradual decrease (Figure 1A). Before BCG vaccination (baseline time point), there were already a proportion of IFN-γ (69%), IL-5 (55%) and IL-13 (36%) responders to PPD (Figure 1C).

To determine whether BCG vaccination influenced the polyclonal non-specific responses as well, cytokine production to PHA was measured. As shown in figure 1B and 1C, the pattern for IFN-γ responses to PHA was increased significantly from pre vaccination to 2 years of age (p<0.001).

**Figure 2:**
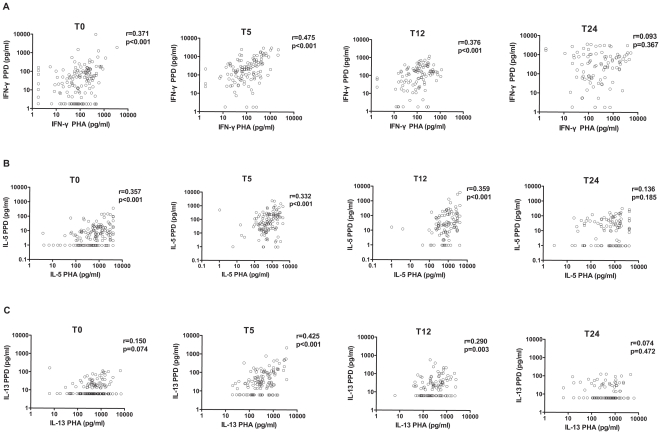
Correlations between PPD and PHA-induced cytokine responses showing IFN-γ (A), IL-5 (B) and IL-13 (C) before BCG vaccination (T0), at 5 months of age (T5), at 1 year of age (T12), and at 2 years of age (T24). . r = Spearman's correlation coefficient, p = *p*-value.

The relations between IFN-γ production in response to PPD and to PHA were analyzed next (Figure 2A). A correlation was seen before vaccination but this was much stronger at 5 months of age which was the first time point after vaccination whereas at two years of age, the IFN-γ production in response to PPD was no longer correlated with IFN-γ response to PHA. Regarding Th2 cytokines (Figure 2B, 2C), before vaccination correlations were weaker than that observed for IFN-γ. After vaccination, at 5 months and 1 year of age, the IL-5 and IL-13 production in response to PPD and PHA were correlated to a better extent. However, as for IFN-γ at 2 years of age the Th2 responses to PPD and PHA were no longer correlated.

### 3.3. Innate immune responses before and after BCG vaccination: early IL-10 and TNF-α production in response to PPD and LPS

**Figure 3:**
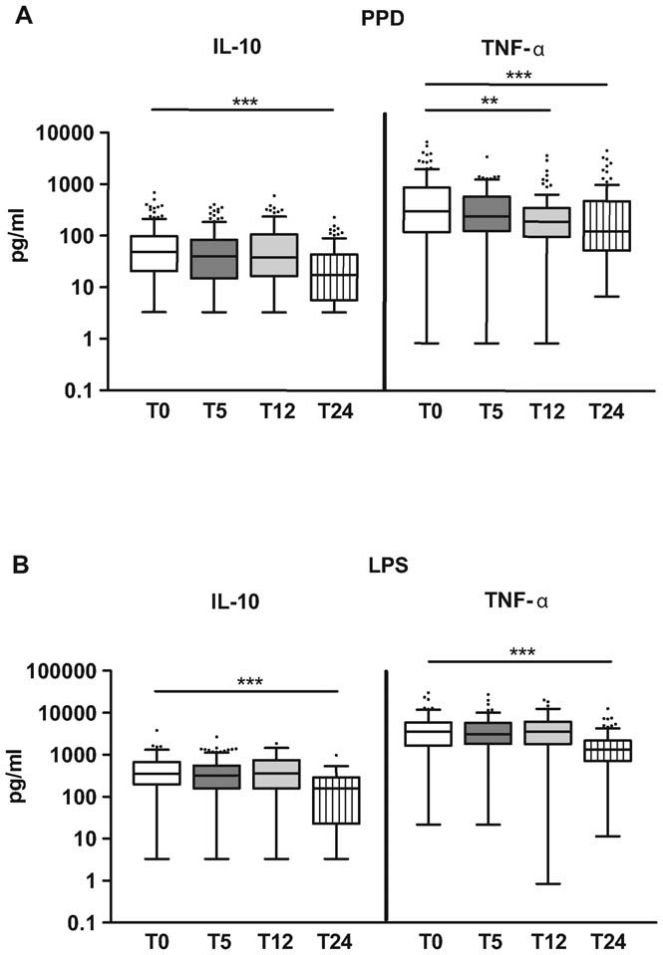
Innate IL-10 and TNF-α responses to PPD (A) and LPS (B). Levels of cytokines at T0 (before vaccination, n = 147 for PPD and n = 101 for LPS), T5 (5 months of age, n = 120 for PPD and n = 117 for LPS), T12 (1 year of age, n = 105 for PPD and n = 103 for LPS) and T24 (2 years of age, n = 98 for PPD and n = 97 for LPS).The horizontal lines in the bars represent the median, with the lower 25% and upper 75% percentiles and extended whiskers the 10% and 90% percentiles. Mann-Whitney test: *0.05>*p*≥0.01; **0.01>*p*≥0.001; ***0.001>*p*.

We were interested in the pro and anti inflammatory innate immune responses and therefore measured IL-10 and TNF-α in day 1 supernatants following stimulation of whole blood with PPD (stimulates TLR2, unpublished data) and LPS (stimulates TLR4). Compared to T0, no increase and even a tendency for a gradual decrease over time was observed for IL-10 response to PPD (T5: p>0.05, T12: p>0.05, T24: p<0.001). Similarly, TNF-α response to PPD decreased over time up to 2 years of age (T5: p>0.05, T12: p = 0.001, T24: p<0.001) (Figure 3A). When responses to a classic innate stimulus, LPS, was examined, patterns similar to innate PPD responses were seen: no increase in IL-10 and TNF-α responses after vaccination, and a significant decrease in both cytokines between T0 and T24 (p<0.001) (Figure 3B). It was also noted that the spontaneous production of IL-10 and TNF-α in day 1 supernatants did not change after vaccination (Table 2).

**Table 2:** Cytokine production in unstimulated (medium) and in PHA, PPD, LPS-stimulated cultures.

Cytokine	Time point	n	Medium	n	PHA	n	PPD	n	LPS
IL-10	T0T5T12T24	14611910598	3.3(3.3–19.1)3.3(3.3–23.4)3.3(3.3–26.1)3.3(3.3–3.3)	14712010598	84.8(42.6–144.4)56.4(31.5–117.2)58.3(30.4–103.4)64.7(40.4–97.5)	14712010598	48.6(20.9–98.7)40.3(15.0–83.9)38.6(16.7–107.0)17.5(5.7–43.9)	10111710397	348.0(195.0–664.7)325.4(161.5–551.9)357.8(159.2–742.1)159.2(22.5–283.9)
TNF-α	T0T5T12T24	14611910598	0.84(0.84–9.7)0.84(0.84–4.7)0.84(0.84–5.2)5.4(0.84–24.0)	14712010598	204.6(63.9–517.3)223.0(78.5–492.8)207.9(87.8–458.5)176.6(58.7–448.3)	14712010598	308.9(122.2–925.6)243.9(126.1–587.8)193.5(98.5–356.2)125.8(53.3–484.5)	10111710397	3528.8(1651.5–5808.7)3054.3(1821.2–5671.1)3553.2(1794.0–6075.0)1312.9(709.2–2173.2)
IFN-γ	T0T5T12T24	14211910397	1.8(1.8–21.8)7.9(1.8–40.6)6.6(1.8–31.0)1.8(1.8–1.8)	14311910397	92.2(35.2–239.3)110.0(41.2–265.6)116.9(41.3–258.7)283.7(89.4–840.8)	14311910397	38.2(4.3–105.6)204.3(72.1–504.1)132.1(40.0–245.6)355.3(63.0–983.5)		
IL-5	T0T5T12T24	14211910397	2.4(1.0–8.6)2.4(1.0–7.5)1.0(1.0–6.1)1.0(1.0–8.1)	14311910397	713.0(223.1–1636.0)554.9(295.7–1421.4)825.4(430.3–2000.0)793.5(262.2–1575.9)	14311910397	5.96(1.0–16.5)64.7(19.3–226.6)27.2(8.4–104.4)19.9(1.0–54.5)		
IL-13	T0T5T12T24	14211910397	6.3(6.3–6.3)6.3(6.3–13.4)6.3(6.3–15.4)6.3(6.3–6.3)	14311910397	497.5(254.6–1164.6)331.3(157.0–803.0)272.5(150.5–645.6)435.9(139.3–1033.4)	14311910397	6.3(6.3–20.3)31.0(14.0–106.6)20.8(6.3–48.8)6.3(6.3–33.2)		

T0: before vaccination (2 months of age), T5: 5 months of age, T12: 1 year of age, T24: 2 years of age. All cytokine levels are expressed in median and interquartile range.

### 3.4. The relationship between maternal parasitic infection status and the cytokine responses to PPD and LPS before vaccination and at different ages after BCG vaccination

Neither filarial infection (data not shown) nor intestinal helminth infection status of mothers had a clear effect on the response of children to PPD in terms of Th1, Th2, pro or anti inflammatory cytokines either at pre or at different ages after vaccination (Table 3).

**Table 3:** Cytokine responses to PPD in children born to mothers with or without intestinal parasitic infection.

Cytokine	Time	n	IH+	n	IH-	*p*	n	IP+	n	IP-	*p*
IL-10	T0T5T12T24	51393435	39.0(14.9–83.6)30.6(13.3–66.3)34.7(14.5–124.4)22.1(10.8–57.9)	88756462	50.6(25.4–103.7)48.3(15.2–93.6)45.2(20.4–120.8)15.2(3.3–37.5)	0.1040.3030.4850.102	39322327	25.6(12.5–49.4)17.2(10.1–45.7)27.8(12.1–81.3)20.4(3.3–45.1)	100827570	62.5(25.6–116.3)52.8(19.2–95.4)49.3(22.5–124.9)17.4(6.5–44.0)	0.0020.0020.0710.942
TNF-α	T0T5T12T24	51393435	200.5(84.8–885.5)184.0(119.1–537.8)237.4(126.3–379.0)160.2(40.3–826.4)	88756462	335.7(149.0–930.1)287.6(126.1–682.1)177.1(86.3–383.8)119.7(55.2–401.9)	0.2280.3480.4380.583	39322327	199.5(77.0–502.5)158.9(78.4–393.3)124.1(42.3–195.3)60.2(30.8–139.0)	100827570	359.4(148.6–1093.7)282.2(136.6–784.3)243.0(119.8–484.8)163.8(80.0–540.6)	0.0050.0200.0110.002
IFN-γ	T0T5T12T24	51383435	21.3(1.8–93.5)159.5(69.9–379.1)114.5(40.2–263.1)563.9(128.4–1143.0)	85756361	50.2(7.4–111.4)210.3(71.3–601.0)158.1(42.5–253.9)273.8(51.1–900.0)	0.0840.4700.7280.104	39312227	18.3(1.8–102.6)140.0(52.2–319.2)163.9(58.0–288.0)171.0(49.6–1057.5)	97827569	48.4(7.1–116.0)234.8(87.9–685.9)132.1(34.1–243.8)360.8(77.6–983.6)	0.1310.0360.4530.483
IL-5	T0T5T12T24	51383435	5.1(1.0–16.4)61.5(12.4–276.8)57.9(15.8–161.6)19.9(1.0–63.6)	85756361	6.5(1–16.8)66.5(22.9–213.6)22.3(7.3–72.1)18.9(1–54.5)	0.6810.7840.0260.618	39312227	6.6(1.0–17.1)47.9(16.9–242.7)28.3(10.9–81.9)13.5(1.0–46.1)	97827569	6.1(1.0–16.5)69.2(20.6–228.7)28.2(8.4–133.0)22.1(1.0–66.3)	0.7260.8900.9900.185
IL-13	T0T5T12T24	51383435	6.3(6.3–18.9)37.7(6.3–113.6)22.1(6.3–58.7)6.3(6.3–42.6)	85756361	6.3(6.3–20.4)30.5(14.1–104.9)20.8(6.3–48.8)6.3(6.3–31.7)	0.9800.6120.5120.863	39312227	6.3(6.3–15.3)25.3(6.3–86.9)9.8(6.3–46.8)6.3(6.3–22.3)	97827569	6.3(6.3–22.8)33.0(16.8–116.7)24.1(6.3–51.2)6.3(6.3–34.2)	0.0320.1900.2630.256

IH: intestinal helminth, IP: intestinal protozoa. T0: before vaccination (2 months of age), T5: 5 months of age, T12: 1 year of age, T24: 2 years of age. *P* values in bold: significant if <0.05. All cytokine levels are expressed in median and interquartile range.

Interestingly, children born to mothers infected with intestinal protozoa in which *Blastocystis hominis* was the predominant species, had consistently lower TNF-α production in response to PPD at T0 (p<0.01), T5 (p = 0.020), T12 (p = 0.011) and T24 (p<0.01) compared to the children born to mothers with no protozoan infections. The same difference was also observed in IL-10 response to PPD at T0 (p<0.01), T5 (p<0.01), and T12 (p = 0.071). Regarding innate responses to LPS, only at T0 children from infected mothers showed significantly lower IL-10 production compared to those from non-infected mothers (p = 0.029). When Th1 and Th2 cytokines as adaptive immune responses were considered, the IFN-γ response to PPD at T5 (p = 0.036) and the IL-13 response to PPD at T0 (p = 0.032) were significantly lower in children born to mothers infected with intestinal protozoa (Table 3). Similar results were found when we compared children from mothers positive for protozoan infection only, with those from mothers negative for both intestinal helminth and protozoan infection (data not shown). However when the children from mothers co-infected with intestinal helminths and protozoa were compared to those from mothers negative for both, the differences became less significant especially after vaccination (Supporting Table 1). Although all findings show that maternal intestinal parasites may influence the degree of child's cytokine production, the pattern of change in any cytokine pattern over time was not affected (data not shown).

### 3.5. Immune responses to PPD and the presence or absence of BCG scar at 4 years of age

**Figure 4:**
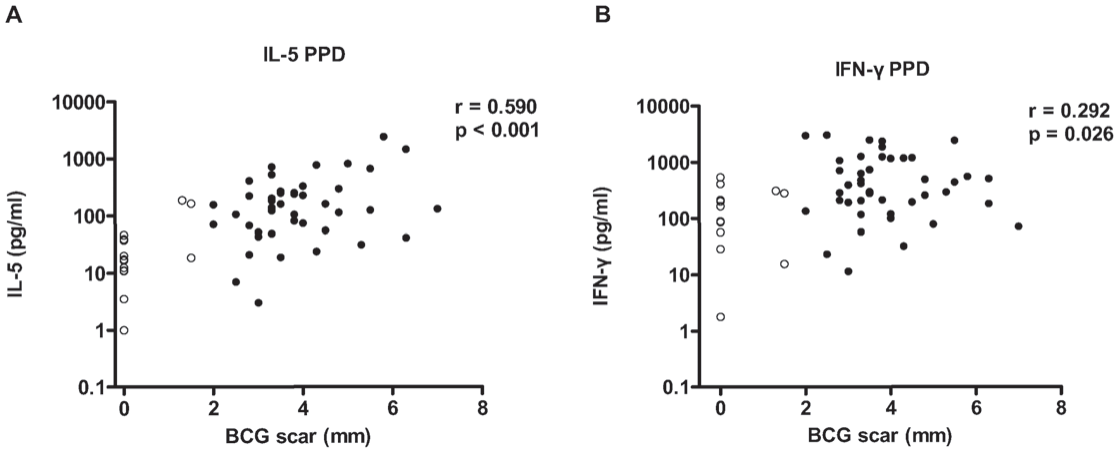
Correlations between PPD-stimulated adaptive cytokine responses and BCG scar size at 5 months of age. IL-5 (A) and IFN-γ (B) responses to PPD and BCG scar size. Open dots represent individuals with BCG scar <2 mm, closed dots represent those with BCG scar > = 2 mm. N = 58 children. r =  Spearman's correlation coefficient, p = *p*-value.

Of sixty six children with complete cytokine data at all time points, there were 58 children with BCG scar measurement at 4 years of age. Since BCG vaccination increased the IFN- γ, IL-5 and IL-13 responses to PPD but not the innate responses as shown in IL-10 or TNF-α responses to PPD, we show the production of IFN- γ and IL-5 in 15 vaccinated children with no BCG scar (<2 mm) and 43 children with a positive BCG scar (≥2 mm) (Figure 4A, 4B). It is clear that some of the children with no BCG scar were still producing cytokines in response to PPD. The cytokine production at 5 months of age, correlated significantly with BCG scar size measured at 4 years of age: IL-5 response to PPD (r = 0.590, p<0.001) and a very weak correlation with IFN-γ to PPD (r = 0.292, p = 0.026). The IL-13 responses were also significantly correlated with BCG scar size (r = 0.427, p = 0.001). We also used multiple regression analysis to be able to adjust for confounders. For this we used data of 61 children that had cytokine data at T5 and a positive BCG scar. IL-5 responses to PPD at 5 months of age stayed significant after adjustment for gender, age at BCG vaccination, birth weight, season at birth, place of residence, maternal intestinal helminth and protozoan infection status. IL-5: estimate (SE)  = 0.049 (0.019), p = 0.012, p_adjusted_  = 0.012. However, IL-13 response to PPD became less significant after adjustment. IL-13: estimate (SE)  = 0.057 (0.021), p = 0.010, p_adjusted_  = 0.051.

The cytokine responses to PPD at older age, thus longer after BCG vaccination were weakly associated with BCG scar size at 4 years of age (data not shown).

## Discussion

The present study shows that BCG not only increases specific adaptive responses in terms of Th1 and Th2 cytokines in response to mycobacterial antigens but it also affects non-specific polyclonal responses. However, we did not find any evidence for its ability to influence pro and anti inflammatory innate immune responses as assessed by early IL-10 and TNF-α production in response to PPD or by LPS.

BCG vaccination enhanced Th1 and Th2 cytokine responses to mycobacterial antigen at 5 months of age (around 20 weeks after vaccination) and although the elevated Th1 responses to PPD were maintained up to 2 years of age, the Th2 responses to PPD waned. In line with our results, several studies in Gambian and United Kingdom infants [15], [16] showed an increase in both Th1 and Th2 responses to PPD measured at 2–3 months after BCG vaccination. With respect to the sustained Th1 responses, Lalor and coworkers observed that in UK infants IFN-γ levels in response to PPD decreased from 3 months to 12 months after vaccination [17]; however in our study, IFN-γ responses to PPD remained high at least until 2 years of age. This might indicate a higher exposure to environmental mycobacteria in our study population, which could help maintain the PPD-specific Th1 memory cells. Moreover, in our study 69% of the infants were producing IFN-γ to PPD before vaccination, which again may reflect the high exposure of these infants to mycobacterial derived products in utero and after birth. Early priming to mycobacteria recorded as cord blood lymphocyte responses to PPD, has been shown in newborns from an area in Kenya highly endemic for tuberculosis, while in the same study US infants did not response to PPD [18]. In agreement with a lack of early priming in areas where exposure to tuberculosis and to environmental mycobacteria is low, Lalor and colleagues found no PPD-induced IFN-γ in unvaccinated infants living in the UK [15]. Furthermore, in an older age group of schoolchildren, IFN-γ production to PPD prior to vaccination was higher in Malawi than in the UK where exposure to environmental mycobacteria is lower [19]. However, in addition to exposure to environmental mycobacteria, many other factors which were not accounted for in our study could influence the Th1 responses, such as the nutritional status [20], genetic background [21], and strain of mycobacteria and its relatedness to *M. bovis* BCG or *M. tuberculosis* [19]. The interesting finding that Th2 responses are not sustained, could indicate that the effect of BCG vaccination is different in magnitude or type from that seen upon natural exposure to mycobacteria, which is via the mucosa. It is also possible that IL-5 and IL-13 production is from cells that do not develop a memory response that can be boosted by re-exposure to mycobacteria.

With respect to adaptive immune responses, it was interesting to note that there was some enhancement of polyclonal responses as assessed by IFN-γ and IL-5 production stimulated by PHA. The correlations between responses to PHA and PPD were strongest at 5 months and 1 year of age but not at later age, which might suggest that BCG can have an effect on cellular immunity beyond that to mycobacterial antigen.

We found no significant increase in IL-10 and TNF-α responses to PPD following BCG vaccination. It should be noted that IL-10 and TNF-α were measured at day 1 post stimulation to give us an insight into early innate responses, which are highest in day 1 supernatants. It has been shown that mycobacteria and its components [22] as well as PPD (our unpublished data indicates that PPD activates TLR2 transfectants) can stimulate innate immune responses through engagement of Toll-Like Receptors such as TLR2 and for some, TLR4, on antigen presenting cells. Almost all children produced IL-10 and TNF-α in response to PPD prior to vaccination and this did not change after vaccination with PPD. The lack of an effect of BCG on innate IL-10 and TNF-α production was confirmed by analyzing responses to LPS. The question whether BCG affects the maturation of innate immune responses in vivo needs to be answered when considering the non-specific beneficial effects of BCG. Our results of BCG vaccination here would argue against any change in the innate IL-10 and TNF-α responsiveness to PPD. However for a formal proof, it is essential to identify which cells are producing the IL-10 and TNF-α and study their dynamics at regular intervals after BCG vaccination, in addition to assessing the expression levels of TLRs and their downstream signaling after delivering the BCG vaccine.

In contrast to the study of Malhotra and coworkers [11], we did not find differences between cytokine responses to PPD after BCG vaccination in children born to helminth-infected mothers and those born to helminth-free mothers. The reason for this discrepancy is not clear. One possibility is that instead of peripheral blood mononuclear cells we used whole blood cultures which would mean that relatively lower numbers of PPD-specific memory cells were stimulated.

In our study, *B. hominis* was found to be the predominant species infecting pregnant mothers, as has been found in Indonesian adults working in Taiwan [25]. Similar to other intestinal protozoa, the presence of *B. hominis* can be associated with poor hygiene and contamination of water and food [26]. So far there have been few studies investigating the early priming of human immune responses by intestinal protozoa and the impact on responses to bystander antigens. One study by Kirch and coworkers showed the production of IgA against *Entamoeba histolytica* antigen by cord blood mononuclear cells of neonates born to seropositive mothers, implying that in utero sensitization by antigens from this intestinal protozoa can occur [27]. Here we show that the presence of intestinal protozoan infection with *B. hominis* as the predominant species in pregnant mothers can dampen the innate and adaptive responses to PPD. Earlier studies have shown that *B. hominis* infection can be associated with impaired intestinal permeability [28] as well as lower total leucocyte and neutrophil count [29]. Whether these may explain the effect seen on the cytokine production in the present study, for *B. hominis* alone or in combination with other pathogens/factors, would need to be investigated further.

Finally, many studies examining the mechanisms behind tissue fibrosis and remodeling have indicated the involvement of Th2 responses in pathogen or chemical induced injury [30,31], which by stimulating collagen formation might initiate the repair processes [32]. However the role of Th2 responses in scar formation induced by BCG has not been studied before. Here we show a strong association between IL-5 or IL-13 responses to PPD early after BCG vaccination and scar formation at 4 years of age when scar formation is thought to be stabilized. A study done by Elliott and colleagues found that IL-5 response to culture filtrate protein of *M. tuberculosis* was correlated with BCG scar size at one year of age especially in infants from hookworm infected mothers [12]. Here we extend the analyses, showing that Th2 responses are indeed correlated with the scar size when it has stabilized at 4 years of age. Although this might be considered a reflection of an overall strong immune responses to BCG vaccination, the fact that there was not a strong correlation with PPD stimulated IFN-γ responses after vaccination, would argue for a selective role of type-2 cytokines stimulated by BCG in scar formation. This could mean that studies assessing scar size are looking more at an early Th2 response induced by BCG rather than generally accepted Th1 responses. Our finding needs to be confirmed in other studies to allow any firm conclusions to be drawn.

In summary, this study has demonstrated the induction of both antigen specific Th1 (long term) and Th2 (short term) cytokine responses, with BCG scar formation associated more strongly with a Th2 cytokine response early after vaccination. Although, the innate IL10 and TNF-α responses were not affected by BCG vaccination, there was some indication of enhancement of adaptive responses beyond PPD after vaccination. These studies need to be taken further by a more in- depth analysis of the immunological changes at the innate and the adaptive immune system in order to be able to understand the non-specific effects of BCG on mortality and morbidity found in epidemiological studies.

## Supporting Information

Table S1 Cytokine responses to PPD in children born to mothers either co-infected with intestinal helminths and protozoa or negative for both.
(0.06 MB DOC)
