# Correction: A Longitudinal Study of BCG Vaccination in Early Childhood: The Development of Innate and Adaptive Immune Responses

**DOI:** 10.1371/annotation/81a1f333-7fb3-4afd-b158-94774d5522fa

**Published:** 2013-12-17

**Authors:** Yenny Djuardi, Erliyani Sartono, Heri Wibowo, Taniawati Supali, Maria Yazdanbakhsh

A reference was excluded from the published reference list. This reference should have been numbered 12 and as a result of this error all subsequent references and citations were incorrectly labeled. Please refer to this document for correct in-text citations and updated references: http://www.plosone.org/corrections/pone.0014066.001.cn.doc

The additional reference is: Yazdanbakhsh M (1999) Common features of T cell reactivity in persistent helminth infections: lymphatic filariasis and schistosomiasis. Immunol Lett 65: 109-115. S0165-2478(98)00133-3 [pii]. 

