# Supplementary material for: A Longitudinal Study of BCG Vaccination in Early Childhood: The Development of Innate and Adaptive Immune Responses
Source: PLoS One. 2010 Nov 19;5(11):e14066. doi: 10.1371/journal.pone.0014066 (PMC2988815; doi:10.1371/journal.pone.0014066)
Supplement: Table S1 — Cytokine responses to PPD in children born to mothers either co-infected with intestinal helminths and protozoa or negative for both. (0.06 MB DOC) [file pone.0014066.s001.doc]

**Table S1. Cytokine responses to PPD in children born to mothers either co-infected with intestinal helminths and protozoa or negative for both.**

| **Cytokine** | **Time point** | **n** | **IH+ IP+** | **n** | **IH- IP-** | ***p*** |
| --- | --- | --- | --- | --- | --- | --- |
| **IL-10** | **T0** | 20 | 25.6 (10.4 – 65.3) | 69 | 64.3 (30.2 – 144.2) | **0.011** |
|  | **T5** | 16 | 23.8 (11.9 – 45.7) | 59 | 53.4 (19.6 – 114.9) | **0.037** |
|  | **T12** | 11 | 29.2 (12.1 – 123.2) | 52 | 53.7 (23.9 – 124.8) | 0.258 |
|  | **T24** | 15 | 23.5 (3.3 – 58.5) | 50 | 15.2 (3.3 – 38.3) | 0.463 |
| **TNF-α** | **T0** | 20 | 214.1 (72.0 – 826.1) | 69 | 475.4 (168.4 – 1115.8) | 0.050 |
|  | **T5** | 16 | 148.8 (78.4 – 504.1) | 59 | 334.9 (138.4 – 790.6) | 0.125 |
|  | **T12** | 11 | 153.9 (100.3 – 425.5) | 52 | 223.4 (109.0 – 509.6) | 0.390 |
|  | **T24** | 15 | 70.0 (24.0 – 373.5) | 50 | 158.3 (76.3 – 524.0) | 0.143 |
| **IFN-γ** | **T0** | 20 | 10.4 (1.8 – 40.7) | 66 | 50.9 (9.9 – 117.6) | **0.026** |
|  | **T5** | 15 | 157.2 (60.8 – 319.2) | 59 | 258.3 (88.7 – 678.7) | 0.102 |
|  | **T12** | 11 | 127.6 (59.6 – 344.7) | 52 | 133.6 (40.7 – 246.0) | 0.690 |
|  | **T24** | 15 | 466.8 (49.6 – 1057.5) | 49 | 307.1 (55.4 – 900.0) | 0.800 |
| **IL-5** | **T0** | 20 | 4.1 (1.0 – 10.4) | 66 | 6.4 (1.0 – 16.5) | 0.697 |
|  | **T5** | 15 | 41.3 (12.4 – 242.7) | 59 | 64.7 (20.9 – 203.2) | 0.568 |
|  | **T12** | 11 | 34.0 (14.0 – 102.9) | 52 | 22.8 (6.6 – 98.7) | 0.319 |
|  | **T24** | 15 | 13.5 (1.0 – 49.7) | 49 | 21.3 (1.0 – 60.7) | 0.524 |
| **IL-13** | **T0** | 20 | 6.3 (6.3 – 17.7) | 66 | 6.3 (6.3 – 23.2) | 0.234 |
|  | **T5** | 15 | 21.7 (6.3 – 106.6) | 59 | 30.5 (14.1 – 108.2) | 0.339 |
|  | **T12** | 11 | 13.8 (6.3 – 105.7) | 52 | 23.0 (6.3 – 50.6) | 0.144 |
|  | **T24** | 15 | 6.3 (6.3 – 22.3) | 49 | 6.3 (6.3 – 31.7) | 0.392 |

IH: intestinal helminth, IP: intestinal protozoa. T0: before vaccination (2 months of age), T5: 5 months of age, T12: 1 year of age, T24: 2 years of age. All cytokine levels are expressed in median and interquartile range. *P* values in bold: significant if < 0.05.
